# Identification of DNA-Binding Proteins Using Mixed Feature Representation Methods

**DOI:** 10.3390/molecules22101602

**Published:** 2017-09-22

**Authors:** Kaiyang Qu, Ke Han, Song Wu, Guohua Wang, Leyi Wei

**Affiliations:** 1 School of Computer Science and Technology, Tianjin University, Tianjin 300350, China; nyqky257248@163.com; 2 School of Computer and Information Engineering, Harbin University of Commerce, Harbin 150028, China; hanke@hrbcu.edu.cn; 3 Center of Potential Illness, Qinhuangdao Hospital of Traditional Chinese Medicine, Qinhuangdao 066001, China; suran.tju@gmail.com; 4 School of Computer Science and Technology, Harbin Institute of China, Harbin 150001, China; ghwang@hit.edu.cn; 5 State Key Laboratory of Medicinal Chemical Biology, Nankai University, Tianjin 300074, China

**Keywords:** DNA-binding protein, mixed feature representation methods, support vector machine

## Abstract

DNA-binding proteins play vital roles in cellular processes, such as DNA packaging, replication, transcription, regulation, and other DNA-associated activities. The current main prediction method is based on machine learning, and its accuracy mainly depends on the features extraction method. Therefore, using an efficient feature representation method is important to enhance the classification accuracy. However, existing feature representation methods cannot efficiently distinguish DNA-binding proteins from non-DNA-binding proteins. In this paper, a multi-feature representation method, which combines three feature representation methods, namely, K-Skip-N-Grams, Information theory, and Sequential and structural features (SSF), is used to represent the protein sequences and improve feature representation ability. In addition, the classifier is a support vector machine. The mixed-feature representation method is evaluated using 10-fold cross-validation and a test set. Feature vectors, which are obtained from a combination of three feature extractions, show the best performance in 10-fold cross-validation both under non-dimensional reduction and dimensional reduction by max-relevance-max-distance. Moreover, the reduced mixed feature method performs better than the non-reduced mixed feature technique. The feature vectors, which are a combination of SSF and K-Skip-N-Grams, show the best performance in the test set. Among these methods, mixed features exhibit superiority over the single features.

## 1. Introduction

DNA-binding proteins are a significant component of living organisms, including prokaryotes and eukaryotic proteomes, such as plant mitochondria [1], human bodies, etc. This protein can bind to DNA and play a vital role in various biological activities [2], such as DNA replication, transcription, recombination, DNA repair, and so on [3,4]. The DNA-binding proteins in plant mitochondria may influence transcription [1]. Histone is a classic type of DNA-binding protein that can help in packaging chromosomal DNA into a compact structure [5]. The DNA-cutting enzyme is a type of DNA-binding protein that can recognize and cut a particular sequence [5]. The single-stranded DNA-binding protein can bind to single-strand DNA to protect it from inappropriate reactions [6,7].

Given the importance of DNA-binding proteins, their recognition and classification have received high concerns. DNA-binding proteins are mainly identified through biological experiments, such as filter-binding assays, genomic analysis, micro-matrix, and chromosomal immunoprecipitation reactions [8]. These experimental methods can provide detailed information for DNA-binding proteins. However, because of the rapidly increasing number of protein sequences, these experimental methods may cost considerable amount of money and time. Therefore, fast and accurate methods must be developed for predicting DNA-binding proteins [9]. Prediction methods that use machine-learning algorithms have recently attracted attention. The machine-learning algorithms have feature representations of proteins, which can help in building classification models and automatically identifying whether the proteins are DNA-binding or not. In the last few decades, many machine-learning methods have been developed for predicting DNA-binding proteins. These methods are divided into two types, namely, sequence-based and structure-based methods.

Sequence-based methods are based only on the protein sequence information. In these methods, the features are extracted by using sequence information, such as amino acid composition and amino acid amount, without considering any structural information [10]. Hence, these methods are highly efficient and useful in predicting large-scale protein sequence datasets [8]. For example, Szilágy and Skolnick [11] predicted DNA-binding proteins according to the amino acid composition through logistic regression by considering the relative proportions of amino acids, the asymmetric spatial distribution of amino acids, and the dipole moment of the molecule. Kumar et al. [12] classified DNA-binding proteins by using a support vector machine (SVM) and by coding the features from evolutionary information. Their study is the first to use position-specific scoring matrix (PSSM) profiles, which include evolutionary information, in predicting DNA-binding proteins. Lin et al. [13] combined the features with a general form of pseudo-amino acid composition using a grey model and a random forest classifier and developed a method with high accuracy rate and is less time consuming. Zou et al. [14] reported a synthesis feature analysis of DNA-binding protein with SVM, thereby opening the possibility of effectively developing a full set of information based on the different scales of protocol sequences and using of SVM integration to accurately predict DNA-binding proteins.

Structure-based feature representation methods use structural and sequence information to identify proteins [10]. For instance, Shanahan et al. [15] focused on the solvent-accessible structural motif and positive electrostatic potential, whereas Bhardwaj et al. [16] studied the surface and overall composition, overall charge, and positive potential patches on the protein surface. Both methods can achieve good results. Under certain conditions, the accuracy can reach 90% for a dataset that contains 121 DNA-binding proteins and 238 non-binding proteins [16]. Cai et al. [17] focused on three structural motifs, namely, helix-turn-helix, helix-hairpin-helix, and helix-loop-helix, and achieved a prediction accuracy of 91.1% under certain conditions, indicating the efficiency of their method in determining the proteins [17].

However, structure-based methods are time consuming. Therefore, these methods are only applicable to small-scale datasets. And sequence-based methods ignore the structural relationship and the physical and chemical properties of proteins. Thus, these methods cannot achieve high accuracy. As previously mentioned, studying an efficient feature representation method is a difficult task.

**Figure 1:**
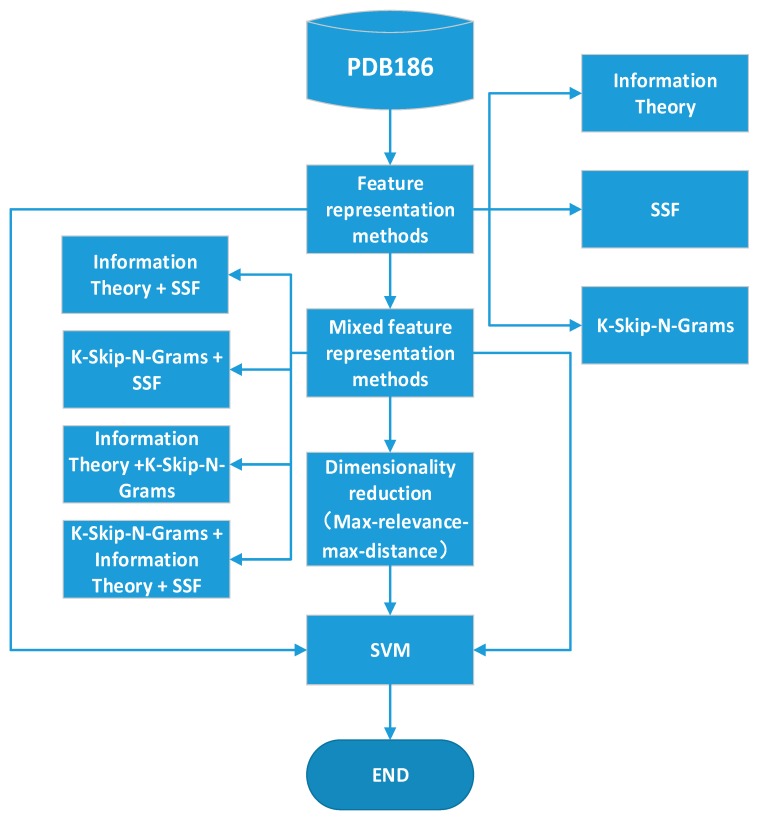
Overview of the paper framework for a DNA-binding protein classifier. First, the protein sequences are represented by Information Theory, SSF, and K-Skip-N-Grams. Then, three methods are combined. Finally, max-relevance-max-distance (MRMD) is used to reduce the dimensions. The support vector machine is used to classify the features which generated by the above three steps, respectively.

This paper uses mixed feature representation with the best performance according to the experimental results. The method entails the following main steps. The protein sequences are represented by three feature representation methods, and SVM is used to build predictive models. These three methods are combined, and max-relevance-max-distance (MRMD) is used to reduce the dimension of features. The mixed feature representation is finally tested through an experiment. Figure 1 shows the experimental process in this paper.

## 2. Methods

### 2.1. Dataset

PDB186 [8] serves as the experimental dataset in this study. PDB186 contains 186 protein sequences with equal numbers of DNA-binding and non-DNA-binding types. Among these sequences, 80% are randomly selected as the training set, whereas the remaining 20% serve as the test set. Therefore, the training set has 148 protein sequences with equal numbers of DNA-binding and non-DNA-binding types, whereas the test set has 38 protein sequences with equal numbers of DNA-binding and non-DNA-binding types.

### 2.2. Classifier

SVMs are built on limited sample learning and provide good results in classifying two categories [18,19,20,21,22,23,24].

In the low-dimensional space, a plane that can correctly partition all data is called a hyperplane. When the distance between two different vectors closest to a plane is at a maximum, the plane is called the optimal hyperplane. The nearest sample from the hyperplane is called the support vector.

A hyperplane can be determined according to a set of support vectors. The hyperplane can be expressed as [25]:

(1) ω·x+b=0

where *x* is the point on the plane and ω is the weight vector perpendicular to the hyperplane. For a two-classification problem, the classification interval size is $\frac{2}{\left|\left|\omega \right|\right|}$. The problem is transformed into the minimum value of $\frac{1}{2}{\left|\left|\omega \right|\right|}^{2}$ under the constraint condition to maximize the classification interval. However, not all problems are linearly separable.

For the non-linearly separable problem, the data from the low-dimensional feature space must be transformed into the high-dimensional feature space through non-linear mapping function. This complicated operation can be avoided by using an efficient kernel function [25].

Different functions have different influences on classification. Gaussian function has a better effect on classification and can be applied in complex data. Therefore, this paper uses the Gaussian function as a kernel function. The formula is as follows:

(2) $$K\left(x_{1},x_{2}\right)=\exp\left(-\frac{{\left|\left|x_{1}-x_{2}\right|\right|}^{2}}{2\sigma^{2}}\right)$$

### 2.3. Single Feature Representation Methods

Three unique features, namely, Information Theory [26], SSF [27], and K-Skip-N-Grams [26] are used in this study.

#### 2.3.1. Information Theory (IT)

Information Theory can produce three feature vectors. The feature representation method mainly expresses the protein sequences through three aspects, namely, Shannon entropy, relative Shannon entropy, and information gain score. The expressions are as follows:

Shannon entropy:

(3) $$\mathrm{SEn}=-\overset{20}{\underset{i=1}{\sum }}p_{i}\log_{2}\left(p_{i}\right)$$

where *p_i_* represents the frequency in which the amino acid *i* appears in the sequence.

Relative Shannon entropy:

(4) $$\mathrm{RSEn}=\overset{20}{\underset{i=1}{\sum }}p_{i}\log_{2}\left(\frac{p_{i}}{p_{0}}\right)$$

where *p*_0_ is the uniform distribution of the amino acid type.

Information gain score:

(5) IGS=SEn−RSEn

#### 2.3.2. Sequential and structural features (SSF)

A total of 473 feature vectors can be generated using SSF.

(a) Based on PSI-BLAST

According to PSSM, 20 features were extracted using the following formula [27,28,29]:

(6) $$FV_{PSSM}=\left\{\bar{S_{j}}=\frac{1}{L}\overset{L}{\underset{i=1}{\sum }}S_{i,j}|j=1,2,\cdots ,20\right\}$$

where $\bar{S_{j}}$ indicates the average score of the amino acid residue at each position of the sequence *S* as mutated by the amino acid residue *j* during evolution.

PSSM is subsequently transformed into a consistent sequence that contains rich evolutionary information. The formula is as follows:

(7) $$S_{i,j}^{\prime }=2^{S_{i,j}\times p_{j}}$$

(8) $$S_{i}=argmax\left\{S_{i,j}^{\prime }:1\leq j\leq 20\right\}\;\left(1\leq i\leq L\right)$$

where *p_j_* represents the frequency in which the amino acid *j* appears in the Protein Data Bank (PDB) and argmax {} denotes the maximum value.

In this step, the *n*-gram features are extracted by using the *n*-gram model. A total of 420 features are obtained and correlated with weights using the formula:

(9) $$FV_{consemus}=\left\{\frac{20}{420}FV_{1-gram},\frac{400}{420}FV_{2-gram}\right\}$$

In summary, 440 feature vectors are obtained based on PSI-BLAST.

(b) Based on PSI-PRED

Protein sequences are extracted according to the secondary structure sequence, and six features are obtained from these sequences. First, three position-specific structural features can be obtained according to the distribution of three spatial types in the secondary structure sequence $S_{str}$. , which is segmented to obtain sequence $S_{seg}$ that only contains *H* fragments (denoted as α) and *E* fragments (expressed as β). The lengths of the continuous spatial structure subunits, *E* and *H*, are obtained. Finally, the frequency in which the fragment βαβ appears in the structural sequence is obtained from $S_{seg}$.

According to the structural probability matrix, the protein sequence is extracted. First, the global features are represented according to the following formula:

(10) $$FV_{global}^{n}=\left\{\frac{1}{L}\overset{L}{\underset{i=1}{\sum }}\left(\overset{n}{\underset{j=1}{\prod }}P_{i,k_{j}}\right),\;j=1,\;2,\cdots ,n;\;k_{j}=1,\;2,\;3\right\}.$$

The number of elements $FV_{global}^{n}\;$ is 3*^n^*, and the number of features depends on the indeterminate *n*.

The structural probability matrix is divided into λ parts according to the rows, and the local information features are extracted according to the following formula:

(11) $$FV_{local}^{n}=\left\{\frac{1}{L\left(m\right)}\overset{index\left(m\right)+L\left(m\right)}{\underset{i=index\left(m\right)}{\sum }}\left(\overset{n}{\underset{j=1}{\prod }}P_{i,k_{j}}\right),\;j=1,\;2,,n;k_{j}=1,\;2,\;3\right\}$$

The local information features can be obtained by combining the features extracted from all sub-matrixes.

$\;\left(\lambda \times 3^{n}+3^{n}+6\right)$ eigenvectors are generated according to PSI-PRED.

Upon combining PSI-BLAST and PSI-PRED, we obtain $\left(20+420+\lambda \times 3^{n}+3^{n}+6\right)$ features [27]. Experiments show that the best performance is obtained at λ = 8 and *n* = 1 [27]. Therefore, the feature representation method can acquire 473 features.

#### 2.3.3. K-Skip-N-Grams (KSNG)

A total of 400 features can be obtained based on K-Skip-N-Grams.

The method sets a fragment with *n* amino acids, which are greater than or equal to 0 and less than or equal to *k* in the protein sequences. In this feature representation method, the eigenvector set is calculated as follows:

(12) $$S_{skipgram}=\left\{\overset{k}{\underset{d=0}{U}}skip\left(DT=d\right)|d=0,\;1,\;2,\cdots ,k;k\leq \frac{l_{min}}{m-1}\right\}$$

where *l_min_* represents the length of the shortest amino acid sequence.

(13) $$FV_{skipgram}=\left\{\frac{N\left(a_{m_{1}}a_{m_{2}}\cdots a_{m_{n}}\right)}{N\left(S_{skipgram}\right)}|1\leq m_{1}\leq 20,\cdots ,1\leq m_{n}\leq 20\right\}$$

where $N\left(S_{skipgram}\right)$ represents the number of elements in the $S_{skipgram}$ set, $a_{m_{1}}a_{m_{2}}\cdots a_{m_{n}}$ represents a sub-sequence fragment including *n* amino acids, and $N\left(a_{m_{1}}a_{m_{2}}\cdots a_{m_{n}}\right)$ represents the number of amino acid sequence fragments in the set $S_{skipgram}$. Thus, the protein sequence can be transformed into a set that includes 20*^n^* features. In this paper, the value of *n* is 2.

### 2.4. Mixed Feature Representation Methods and Feature Selection

Given the limitations of the three feature representation methods, this paper considers the mixed feature representation methods to ensure that each new feature vector contains various features. In this paper, three feature representation methods, namely, Information Theory, SSF, and K-Skip-N-Grams, are combined to obtain four mixed methods, namely, SSF + K-Skip-N-Grams, Information theory + K-Skip-N-Grams, SSF + Information theory, and SSF + Information theory + K-Skip-N-Grams. The mixed feature representation methods allow the features to represent the protein sequences from many aspects and improve the classification effect.

However, the mixed feature vectors may contain redundant or even contradictory vectors. Hence, the dimensions must be reduced. MRMD is the dimension reduction method used in this paper [30]. The basic principle is to determine the feature subset with the largest correlation and the largest distance between the features according to a certain weight ratio. Thus, eight sets of features are obtained from the four mixed methods, thereby producing eight sets of experimental data in this part of the experiment.

## 3. Experiment

### 3.1. Measurement

Four commonly used metrics, such as Sensitivity (SN), specificity (SP), accuracy (ACC), and Matthew’s correlation coefficient (MCC), have been widely used for performance evaluation. Thus they are employed in this work and are calculated for test set validation as follows:

(14) SN=TP/TP+FN

(15) SP=TN/TN+FP

(16) $$\mathrm{ACC}=\frac{TN+TP}{TN+FP+TP+FN}$$

(17) $$\mathrm{MCC}=\frac{\left(TP\times TN\right)-\left(FP\times FN\right)}{\sqrt{\left(TP+FP\right)\left(TP+FN\right)\left(TN+FP\right)\left(TN+FN\right)}}$$

where true positive (*TP*) represents the number of identified DNA-binding proteins in the positive sequences, false positive (*FP*) represents the number of predicted positive samples in the negative set; true negative (*TN*) represents the number of correctly sorted non-DNA-binding protein in the negative sequences; and false negative (*FN*) represents the number of identified negative samples in the positive set.

This paper uses test set validation and 10-fold cross-validation, which divides the data into 10 groups to obtain 10 classification results. An evaluation value can be obtained from the average accuracy rate of the 10 categories. Test set validation randomly divides the dataset into two groups, namely, a training set and a test set. First, the training set is used to train the SVM. Upon establishing the classification model, it is subsequently used for the test set.

### 3.2. Performance of Different Features

We use the single feature representation method to classify the protein sequences, and the results are shown in Table 1.

According to Table 1, SSF has the best accuracy at 66.22% and 78.95%, followed by K-Skip-N-Grams and Information theory.

According to the experimental results, the best classification method includes physical and chemical properties, structural information, and evolutionary information.

### 3.3. Performance of the Mixed Features

The three feature extraction methods are combined in various ways to obtain new feature vectors. The obtained feature vectors are used for the experiment in the following two steps. First, a SVM classifies the mixed feature vectors. Second, a SVM classifies the new feature vectors with dimensions reduced by MRMD. The results are in Table 2.

According to the data in the above Table 2, the accuracy of 10-fold cross-validation after using MRMD dimension reduction is basically higher than that without dimension reduction. However, the combination of Information theory and K-Skip-N-Grams is a special case; the result of dimension reduction is worse than that of non-dimension reduction. The combination of the three methods after dimension reduction has the best accuracy rate based on 10-fold cross-validation, whereas the combination of SSF and K-Skip-N-Grams has the best classification accuracy based on the test set validation.

### 3.4. Comparison with State-of-the-Art Methods

This paper compares the different combinations of feature representation with existing feature representations, such as PseDNA-Pro [31], DNAbinder (P400) [12], DNAbinder (P21) [12], DNA-Prot [32], and iDNA-Prot [13]. In this section, the PDB1075 dataset [10] is used and analyzed through jackknife validation. The experimental data are derived from the study of Liu [31].

According to Table 3, the methods proposed in this paper can achieve high accuracy, especially after dimension reduction. The mixed features compensate for the shortcomings of the individual methods. By reducing the dimensions with MRMD, the redundant vectors are removed, and the efficiency of classification is improved.

However, the experimental results are not particularly ideal. The possible reasons are as follows:

(1) The dataset is extremely small.

In this paper, we only use the PDB186 dataset. The training set contains 74 positive cases and 74 negative cases, whereas the test set contains 19 positive cases and 19 negative cases. The small dataset affects the experimental results.

(2) The combination of feature representation method is extremely simple.

This paper does not consider the differences among the different feature representation methods and the proportion in the classification. Future studies should focus on the corresponding weights for different feature representation methods and on increasing the proportion of important features to improve the accuracy of classification.

### 3.5. Comparison with Other Classifiers

A random forest [33] is used as a classifier to compare with a SVM and explore the effect of the classifier type on DNA-binding protein classification.

The PDB186 dataset is classified and identified by using a random forest as a classifier and Information Theory, SSF, K-Skip-N-Grams, SSF + K-Skip-N-Grams, Information theory + K-Skip-N-Grams, SSF + Information theory, and SSF + Information theory + K-Skip-N-Grams as feature representation methods. The results are in Table 4 and Table 5.

When a random forest is used as the classifier, the accuracy of mixed feature representation methods is higher than that of the single feature representation methods (Table 4 and Table 5). The classification accuracy of the dimension-reduced feature vectors is better than that of non-dimensionally-reduced feature vectors. The combination of SSF, Information theory, and K-Skip-N-Grams has the lowest accuracy; however, this value is improved as compared with that of single K-Skip-N-Grams.

**Figure 2:**
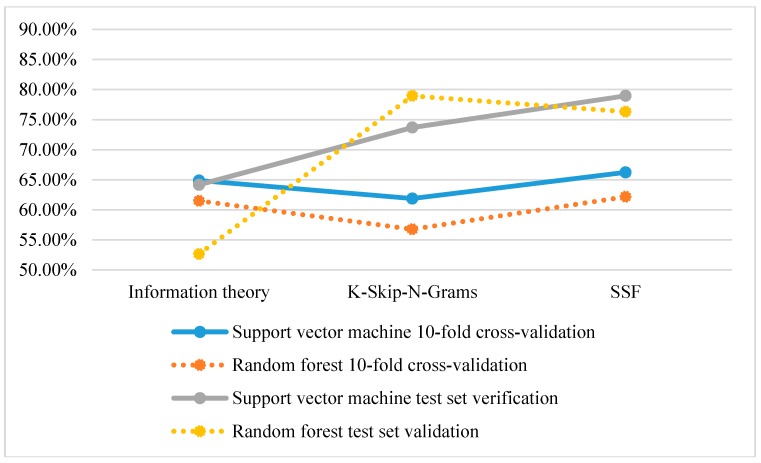
Comparison of the classification accuracy of single feature representation methods.

From Figure 2, the classification accuracy of using SVM is higher than that of using a random forest based on 10-fold cross-validation and test set verification. However, in the test set validation of K-Skip-N-Grams, the accuracy of using a random forest is higher than that of using SVM.

**Figure 3:**
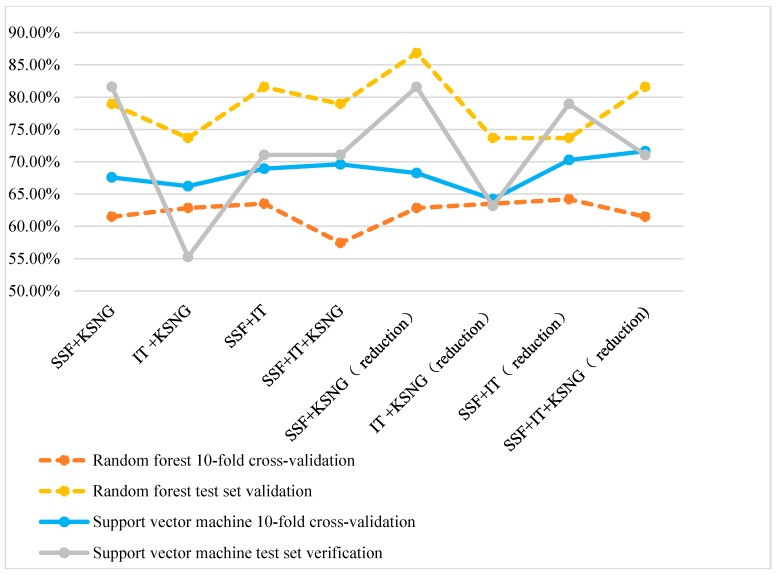
Comparison of the accuracy of multiple feature representation methods.

From Figure 3, the accuracy of using a SVM is higher than that of using random forest during 10-fold cross-validation. However, random forest classification is superior to SVM during test set verification.

## 4. Conclusions

This paper uses three feature representation methods and four mixed feature representation methods. The protein sequences in PDB186 are extracted and subjected to classification experiments. The accuracy rate is generally improved after combining the feature methods. Moreover, the dimension-reduced features show better performance than non-reduction mixed features. In comparison, the accuracy in SVM classification is higher than with random forest for 10-fold cross-validation. However, for the test set, the classification effect of mixed feature representation method in a random forest classifier is better than that with a SVM. Currently, a webserver is freely available at http://server.malab.cn/IKP-DBPPred/index.jsp.

Future studies should focus on the corresponding weights of different feature representation methods and on increasing the proportion of an important feature to improve the accuracy of classification.

## Figures and Tables

**Table 1:** The results of using single feature representation methods and the PDB186 dataset.

Method	Ten-Cross Validation Accuracy (%)	Test Set Validation Accuracy (%)			
		SN	SP	MCC	ACC
Information theory	64.86	68.42	57.89	0.26	64.16
K-Skip-N-Grams	61.86	68.42	78.95	0.48	73.68
SSF	66.22	73.68	84.21	0.58	78.95

**Table 2:** Results of protein classification based on multiple features and PDB186 dataset.

Method	Non-Dimensionality-Reduction		Dimensionality-Reduction	
	Ten-Cross Validation Accuracy (%)	Test Set Validation Accuracy (%)	Ten-Cross Validation Accuracy (%)	Test Set Validation Accuracy (%)
SSF + K-Skip-N-Grams	67.57	81.58	68.24	81.58
Information theory + K-Skip-N-Grams	66.22	55.26	64.19	63.16
SSF + Information theory	68.92	71.05	70.27	78.95
SSF + Informationtheory + K-Skip-N-Grams	69.59	71.05	71.62	71.05

**Table 3:** Accuracy of existing feature representation methods using PDB1075 dataset.

Method	References	ACC (%)	MCC	SN (%)	SP (%)
SSF + Informationtheory + K-Skip-N-Grams (reduction)	This paper	77.43	0.55	77.84	77.05
SSF + Informationtheory + K-Skip-N-Grams	This paper	75.19	0.51	76.88	73.59
PseDNA-Pro	[31]	76.55	0.53	79.61	73.63
DNAbinder (P400)	[12]	73.58	0.47	66.47	80.36
DNAbinder (P21)	[12]	73.95	0.48	68.57	79.09
DNA-Prot	[32]	72.55	0.44	82.67	59.76
iDNA-Prot	[13]	75.40	0.50	83.81	64.73

**Table 4:** The PDB186 is classified by a random forest and single feature representation.

Method	Ten-Cross Validation Accuracy (%)	Test Set Validation Accuracy (%)
Information theory	61.49	52.63
K-Skip-N-Grams	56.76	78.95
SSF	62.16	76.32

**Table 5:** PDB186 is classified by a random forest using mixed feature representations.

Method	Non-Dimensionality-Reduction		Dimensionality-Reduction	
	Ten-Cross Validation Accuracy (%)	Test Set Validation Accuracy (%)	Ten-Cross Validation Accuracy (%)	Test Set Validation Accuracy (%)
SSF + K-Skip-N-Grams	61.49	78.95	62.84	86.84
Information theory + K-Skip-N-Grams	62.84	73.68	63.51	73.68
SSF + Informationtheory	63.51	81.58	64.19	73.68
SSF + Informationtheory + K-Skip-N-Grams	57.43	78.95	61.49	81.58
